# Improving photosynthesis to increase grain yield potential: an analysis of maize hybrids released in different years in China

**DOI:** 10.1007/s11120-021-00847-x

**Published:** 2021-05-25

**Authors:** Yanyan Yan, Peng Hou, Fengying Duan, Li Niu, Tingbo Dai, Keru Wang, Ming Zhao, Shaokun Li, Wenbin Zhou

**Affiliations:** 1 Institute of Crop Sciences, Chinese Academy of Agricultural Sciences, Beijing, 100081 China; 2 Key Laboratory of Crop Physiology Ecology and Production Management of Ministry of Agriculture, Nanjing Agricultural University, Nanjing, 210095 China

**Keywords:** Grain yield, Photosynthesis, Density-tolerance, Planting density, Maize

## Abstract

In this work, we sought to understand how breeding has affected photosynthesis and to identify key photosynthetic indices that are important for increasing maize yield in the field. Our 2-year (2017–2018) field experiment used five high-yielding hybrid maize cultivars (generated in the 1970s, 2000s, and 2010s) and was conducted in the Xinjiang Autonomous Region of China. We investigated the effects of planting density on maize grain yield, photosynthetic parameters, respiration, and chlorophyll content, under three planting density regimens: 75,000, 105,000, and 135,000 plants ha^−1^. Our results showed that increasing planting density to the medium level (105,000 plants ha^−1^) significantly increased grain yield (Y) up to 20.32% compared to the low level (75,000 plants ha^−1^). However, further increasing planting density to 135,000 plants ha^−1^ did not lead to an additional increase in yield, with some cultivars actually exhibiting an opposite trend. Interestingly, no significant changes in photosynthetic rate, dark respiration, stomatal density, and aperture were observed upon increasing planting density. Moreover, our experiments revealed a positive correlation between grain yield and the net photosynthetic rate (P_n_) upon the hybrid release year. Compared to other cultivars, the higher grain yield obtained in DH618 resulted from a higher 1000-kernel weight (TKW), which can be explained by a longer photosynthetic duration, a higher chlorophyll content, and a lower ratio of chlorophyll *a*/*b*. Moreover, we found that a higher leaf area per plant and the leaf area index (HI) do not necessarily result in an improvement in maize yield. Taken together, we demonstrated that higher photosynthetic capacity, longer photosynthetic duration, suitable LAI, and higher chlorophyll content with lower chlorophyll *a*/*b* ratio are important factors for obtaining high-yielding maize cultivars and can be used for the improvement of maize crop yield.

**Supplementary Information:**

The online version contains supplementary material available at 10.1007/s11120-021-00847-x.

## Introduction

In order to meet the needs of an ever-increasing human population, global crop production rate needs to double by 2050 (2.4% annual growth rate) (Pradhan et al. 2015). However, the average output of major crops increases by only 0.9–1.6% per year, which remains far below the required levels (Ray et al. 2013). This puts a significant pressure on global food security and makes the cultivation of high-yielding crops an agricultural necessity, whereby increasing crop yield potential constitutes a major breeding goal of the modern era (Foley et al. 2011; Evans and Lawson 2020; Phalan et al. 2011; Paz et al. 2020). Maize is a high-yielding C_4_ grain-producing cereal and the second largest crop in the world, representing a central target for improving total crop production capacity (FAO 2017; Ort and Long 2014). The past decades witnessed significant efforts by maize breeders to consistently increase grain yield (Tollenaar 1989; Niu et al. 2013) despite a recent deacceleration, especially in Europe (Foley et al. 2011). Agronomic management, including the application of fertilizers or increasing plant density, affects breeding strategies and plays an important role in the attempt to improve crop yield (Lu and Tian 2017; Mansfield and Mumm 2014). In the specific case of maize, crop production has reached a plateau in several countries, as indicated by little changes in maize grain yield per unit area in China (Gong et al. 2015; Ray et al. 2012). Based on a meta-analysis of 140 published results, Slattery et al. found that fertilizer supply, CO_2_ concentration, and shading have a significant influence on crop yield. Hence, improving photosynthesis can help increasing yield (Slattery et al. 2013). Moreover, and considering that (1) traditional breeding and selection did not focus on photosynthetic efficiency and (2) that CO_2_ concentration is predicted to increase as a result of global climate change, it is necessary to better understand photosynthesis in order to continuously increase yield potential (Long et al. 2006; Raines 2011; Zhu et al. 2010).

A few studies aiming to elevate crop yield have already attempted to manipulate photosynthetic efficiency (Parry et al. 2011; Wang et al. 2018; Li et al. 2020), and previous results suggested that increased grain yield can be achieved by increasing leaf area and the absorption of radiation (Testa et al. 2016; Meena et al. 2021). Furthermore, it has been shown that increasing plant density significantly enhances maize grain yield due to a higher capture efficiency of photosynthetically active radiation and light energy use, improving the photosynthetic capacity of the canopy (Zhang et al. 2014). In fact, a positive correlation between the photosynthetic rate at the canopy level and the productivity of individual plants has been reported across different crops, including C_3_ plants such as wheat, barley, or soybean (Reynolds et al. 2000; Carmo-Silva et al. 2017; Biscoe et al. 1975; Wells et al. 1982), and C_4_ plants including maize and sorghum (Vietor and Musgrave 1979; Peng et al. 1991). However, these results have been challenged and the increase in photosynthetic rates is regarded as slight and not significantly contributing to changes in crop biomass and yield (Chytyk et al. 2011; Sadras et al. 2012; Wu et al. 2019; Leakey et al. 2019). According to Zelitch (1982), the perceived lack of relationship between photosynthesis and production results from an instantaneous measurement of photosynthetic activity at improper developmental stages. Moreover, the experimental conditions in the laboratory do not necessarily reflect natural conditions in the field, and variation at the genetic level might impact photosynthetic efficiency (Reynolds et al. 2000; Zelitch 1982) In summary, the relationship between leaf photosynthesis and yield remains controversial (Sinclair et al. 2019).

The goal of this study was to determine whether maize varieties released in different years show differences in photosynthetic capacity. Furthermore, we aimed at understanding to what extent the ear leaf photosynthesis reflects differences in yield across distinct cultivars. Our research was conducted in the Xinjiang Uygur Autonomous Region, where sufficient sun light, radiation, and a large diurnal temperature range represent adequate environmental factors that allow for the cultivation of high-yield maize crops. In the present study, five maize hybrids released in different years which had created highest yield in China were used to evaluate the role of photosynthesis in determining maize grain yield. To achieve this, we systematically measured maize yield components, leaf area, photosynthetic parameters (including chlorophyll amount), and stomatal status in the maize hybrids grown under different planting densities. In addition, we analyzed the ratio of red to far-red light at the ear position, the chlorophyll fluorescence at 77 K, and the accumulation of biomass, to investigate the physiological mechanisms underlying the production of high-yield maize mediated by photosynthetic activity.

## Materials and methods

### Experimental location and environmental conditions

**Fig. 1:**
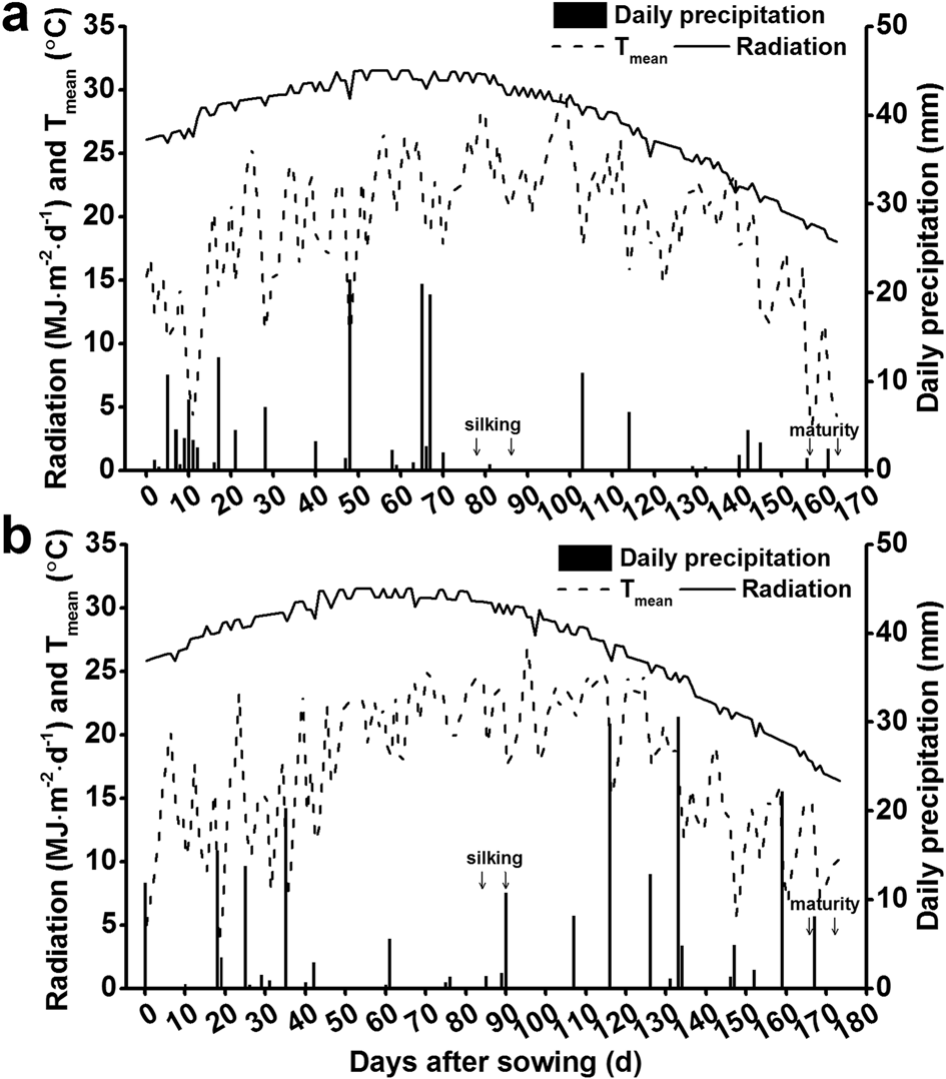
Radiation (MJ m^−2^ day^−1^), daily mean temperature (T_mean_,  °C), and daily precipitation (mm) during the growth period of maize in 2017 (**a**) and 2018 (**b**). The data range of the silking and filling stages includes five varieties that were marked with arrow lines

The field experiments were conducted at Qitai farm (89º 34′ E, 44º 12′ N) during the growing season (April to October) in 2017 and 2018. The experimental site was located in the Xinjiang Uygur Autonomous Region, which is situated in northwestern China, an irrigated spring maize region with an arid continental climate. The annual average precipitation and solar radiation in this region are approximately 269.4 mm and 14.4 MJ m^2^ day^−1^. The mean daily maximum temperature, minimum temperature, and diurnal temperature variation are 13.9 °C, − 0.8 °C, and 14.9 °C, respectively. The climatic conditions of 2017 and 2018 during the maize growing season are shown in Fig. 1. During the growing season, the mean daily maximum temperature, the mean daily minimum temperature, and the mean daily temperature were 25.6 °C, 12.8 °C, and 19.1 °C in 2017, and 24.3 °C, 10.6 °C, and 17.3 °C in 2018. In 2017 and 2018, the total radiation was 4521.9 MJ m^−2^ and 4686.7 MJ m^−2^, and the total precipitation was 175.2 mm and 221.0 mm, respectively.

We used five different maize cultivars in this study, including Zhengdan 958 (ZD958), Xianyu 335 (XY335), Denghai 618 (DH618), Liangyu 66 (LY66), and SC704. The maize hybrids were released at different times over the previous decades (SC670 in 1970s; ZD958, XY335 and LY66 in 2000s; DH618 in 2010s), and each had the highest yield at the Qitai farm in different years (see Table S1). Each cultivar was planted at three planting densities: 75,000 plants ha^−1^ (low density, L), 105,000 plants ha^−1^ (medium density, M) and 135,000 plants ha^−1^ (high density, H). The low density is recommended for most parts of China (Ming et al. 2017; Hou et al. 2020), the medium density is associated with higher yield (Yang et al. 2020), and the high density has been adopted in high-yield experiments (Liu et al. 2017, 2020). We employed similar treatments to the plants in 2017 and 2018. However, there was an unexpected snow at the seedling stage in 2018, resulting in a relatively lower actual density when compared to 2017. Hence, we only used the plots which have proper plant density for downstream experiments. The actual planting density at harvest is shown in Tables 1 and 2. Maize seeds were sown by hand on April 21 and 17, and plants were harvested on October 1 and 14, in the years 2017 and 2018, respectively. In each year, the experimental treatments were arranged in a split–split plot design, where the density and the cultivars were set as the main plot and subplot, respectively, with three experimental replicates performed per treatment. In order to match the local cultivation practices, the plots were set up in a narrow-wide row planting pattern, which consisted of alternating rows 0.4 and 0.6 m apart, with each plot measuring 10 m long × 8 m wide. The seeds were sown under plastic film with a drip irrigation system to prevent drought stress. Sufficient water and fertilizers were applied to prevent deficient water and nutrient stress. Base fertilizers were applied with 150 kg N ha^−1^ as urea, 225 kg P_2_O_5_ ha^−1^ (super phosphate), and 75 kg K_2_O ha^−1^ (potassium sulfate) prior to sowing. An additional 300 kg N ha^−1^ was applied during the growing season to prevent nutritional stress. The accumulation of weeds, and the management of diseases and pests were implemented following the protocols of Yang et al. (2019).

**Table 1:** Grain yield and yield components, total biomass, and HI of five maize hybrids cultivated under different densities in 2017 and 2018

Year	Cultivar	Plant density (plant·m^2^)	Grain Yield (Mg·ha^−1^)	Ear No. (10^3^ ha^−1^)	Kernel No. per ear	1000-Kernel weight (g)	Total biomass accumulation (t·ha^−1^)	HI
2017	SC704	7.5 (L)	16.53 ± 0.69e	67.45 ± 5.77i	633.87 ± 22.76a	370.25 ± 16.40bcde	31.63 ± 1.65ef	0.523 ± 0.006cde
		10.5 (M)	18.21 ± 1.98cde	95.52 ± 8.10g	571.47 ± 3.06c	346.65 ± 6.26def	34.24 ± 2.43cde	0.531 ± 0.02cde
		13.5(H)	13.46 ± 0.89f	90.75 ± 6.70g	488.27 ± 18.48de	326.36 ± 34.83f	37.52 ± 2.63bcd	0.359 ± 0.006h
	ZD958	7.5 (L)	16.24 ± 1.60e	90.48 ± 5.02g	474.40 ± 18.07de	381.64 ± 18.35bcd	30.63 ± 0.67ef	0.530 ± 0.043cde
		10.5 (M)	18.73 ± 0.68cd	115.15 ± 5.55de	449.13 ± 17.50ef	364.91 ± 10.14cdef	33.18 ± 1.26de	0.564 ± 0.002c
		13.5 (H)	18.88 ± 1.11cd	140.51 ± 6.79a	421.90 ± 7.80f	342.85 ± 19.27def	34.47 ± 4.79cde	0.552 ± 0.054cd
	XY335	7.5 (L)	19.23 ± 0.63cd	80.45 ± 3.52h	618.27 ± 16.54ab	400.77 ± 21.27bc	37.60 ± 3.19abcd	0.513 ± 0.027de
		10.5 (M)	19.63 ± 1.68cd	105.55 ± 3.24f	511.47 ± 65.26d	368.73 ± 19.52bcde	42.12 ± 4.44a	0.467 ± 0.019fg
		13.5(H)	19.81 ± 1.61cd	130.38 ± 2.04b	418.27 ± 35.72f	373.79 ± 10.63bcde	40.19 ± 3.98ab	0.493 ± 0.009ef
	LY66	7.5 (L)	19.88 ± 0.76c	80.25 ± 2.72h	620.40 ± 17.01ab	406.28 ± 38.18ab	37.43 ± 0.82bcd	0.531 ± 0.009cde
		10.5 (M)	23.92 ± 0.81a	105.33 ± 1.89f	577.05 ± 10.58bc	404.40 ± 15.85abc	38.30 ± 0.14abc	0.624 ± 0.019b
		13.5(H)	17.66 ± 0.96de	120.18 ± 5.36cd	448.27 ± 18.90ef	338.26 ± 26.38ef	39.87 ± 3.04ab	0.444 ± 0.017g
	DH618	7.5 (L)	19.00 ± 0.35cd	92.29 ± 4.39g	485.60 ± 18.12de	441.54 ± 23.13a	27.51 ± 2.00f	0.693 ± 0.044a
		10.5 (M)	22.44 ± 0.93ab	109.13 ± 3.59ef	477.87 ± 13.25de	440.49 ± 12.39a	33.21 ± 1.67de	0.676 ± 0.017a
		13.5(H)	21.82 ± 0.06b	127.77 ± 1.77bc	424.53 ± 6.52f	401.30 ± 6.94bc	38.78 ± 0.89abc	0.563 ± 0.014c
	ANOVA							
	Density (D)		20.855**	252.588**	99.854**	16.303**	17.909**	53.955**
	Variety (V)		28.435**	51.532**	38.839**	18.043**	16.617**	62.212**
	D × V		7.095**	6.171**	6.048**	1.222 ns	2.116 ns	13.419**
2018	SC704	6.3 (L)	15.60 ± 1.14d	60.35 ± 4.21c	744.3 ± 93.60a	369.3 ± 8.59d	24.17 ± 0.99e	0.645 ± 0.023b
		11.3 (H)	16.91 ± 1.42bcd	92.12 ± 8.32a	555.6 ± 55.31cde	325.8 ± 7.60e	37.31 ± 4.89b	0.456 ± 0.033 fg
	ZD958	6.9 (L)	18.65 ± 0.38abc	75.57 ± 2.52b	615.7 ± 40.11bcde	418.4 ± 16.87a	29.94 ± 1.15d	0.624 ± 0.033bc
		9.7 (M)	20.31 ± 2.68a	98.04 ± 2.43a	552.1 ± 65.95de	383.5 ± 17.78bcd	27.91 ± 1.21de	0.726 ± 0.065a
	XY335	7.0 (L)	18.10 ± 1.95abcd	68.63 ± 5.86bc	663.5 ± 19.06b	406.5 ± 11.29ab	36.16 ± 2.90b	0.500 ± 0.017ef
		11.1 (H)	19.43 ± 2.21ab	103.55 ± 16.03a	531.5 ± 16.75e	379.7 ± 16.91cd	46.62 ± 1.74a	0.416 ± 0.034g
	LY66	4.9 (L)	16.44 ± 1.52cd	67.63 ± 5.73bc	640.0 ± 33.26bc	417.2 ± 19.58a	31.21 ± 1.67cd	0.526 ± 0.024e
		7.9 (M)	18.18 ± 1.14abcd	76.35 ± 5.16b	617.7 ± 8.05bcd	398.4 ± 4.12abc	35.39 ± 3.98b	0.516 ± 0.028e
	DH618	7.5 (L)	19.34 ± 1.02ab	75.86 ± 3.26b	626.3 ± 20.10bcd	416.4 ± 5.99a	35.92 ± 1.92b	0.538 ± 0.009de
		9.9 (M)	20.02 ± 1.04a	94.05 ± 3.71a	556.1 ± 33.13cde	390.1 ± 14.43bcd	34.03 ± 1.81bc	0.589 ± 0.025cd
	ANOVA							
	Density (D)		6.69*	90.824**	33.904**	34.123**	34.022**	5.329*
	Variety (V)		6.392**	6.026**	2.343 ns	18.296**	27.59**	38.847**
	D × V		0.129 ns	3.759*	3.178*	0.668 ns	14.453**	20.382**

Maize plants suffered from cold damage at the seedling stage, resulting in a relatively lower actual density in 2018. The mean values indicated within a column and the same site followed by different letters denote significant differences at *p* < 0.05. L indicates plant density ≤ 7.5 plants m^−2^. M denotes plant density > 7.5 and ≤ 10.5 plants m^−2^. H denotes plant density > 10.5 plants m^−2^. The numbers shown in the ANOVA section represent the F values. ns Not significant; * Significant at the 0.05 probability level; ** Significant at the 0.01 probability level; *** Significant at the 0.001 probability level

**Table 2:** Dry matter, yield, and leaf area per plant at silking and maximum leaf area index of five maize hybrids cultivated under different densities in 2017 and 2018

Year	Cultivar	Density	Dry matter per plant (g·plant^−1^)		Yield per plant (g·plant^−1^)	Leaf area per plant (10^3^ cm^2^·plant^−1^)	LAI
			Silking	Maturity			
2017	SC704	7.5 (L)	176.38 ± 11.65a	421.73 ± 21.97b	220.34 ± 9.25b	7.08 ± 0.28abcd	5.31 ± 0.21gh
		10.5 (M)	161.38 ± 11.75ab	326.06 ± 23.15d	173.44 ± 18.81cd	6.98 ± 0.41abcde	7.33 ± 0.43d
		13.5(H)	117.07 ± 13.19e	277.95 ± 19.47ef	99.73 ± 6.61f	5.99 ± 0.36 fg	8.09 ± 0.49cd
	ZD958	7.5 (L)	136.65 ± 9.41cd	408.40 ± 8.99b	216.60 ± 21.42a	7.23 ± 0.74abc	5.42 ± 0.56fgh
		10.5 (M)	139.88 ± 10.57cd	316.00 ± 12.01de	178.34 ± 6.49c	7.70 ± 0.74a	8.08 ± 0.78cd
		13.5 (H)	119.76 ± 2.85e	255.35 ± 35.48f	139.86 ± 8.20ef	6.78 ± 0.47bcdef	9.15 ± 0.64ab
	XY335	7.5 (L)	144.86 ± 10.41cd	501.32 ± 42.50a	256.38 ± 8.41a	6.36 ± 0.55cdefg	4.77 ± 0.42hi
		10.5 (M)	140.46 ± 4.54cd	401.12 ± 42.33bc	186.96 ± 16.04b	6.19 ± 0.31efg	6.50 ± 0.33e
		13.5(H)	120.49 ± 7.86e	297.73 ± 29.47def	146.74 ± 11.94de	6.94 ± 0.09abcde	9.37 ± 0.13a
	LY66	7.5 (L)	174.26 ± 15.37a	499.03 ± 10.90a	265.09 ± 10.16a	7.61 ± 0.42ab	5.71 ± 0.32efg
		10.5 (M)	151.84 ± 9.46bc	364.80 ± 1.35c	227.83 ± 7.68b	7.21 ± 0.59abc	7.57 ± 0.62d
		13.5(H)	115.46 ± 6.86e	295.37 ± 22.56def	130.80 ± 7.12f	6.31 ± 0.07defg	8.52 ± 0.10bc
	DH618	7.5 (L)	131.45 ± 5.37de	366.75 ± 26.70c	253.28 ± 4.67b	5.93 ± 0.27fg	4.45 ± 0.20i
		10.5 (M)	115.62 ± 8.79e	316.32 ± 15.94de	213.73 ± 8.84 cd	5.89 ± 0.38g	6.18 ± 0.40ef
		13.5 (H)	98.70 ± 5.56f	287.24 ± 6.56def	161.63 ± 0.46g	5.78 ± 0.24g	7.81 ± 0.32cd
	ANOVA						
	Density (D)		71.595**	177.332**	329.78**	5.05*	219.478**
	Variety (V)		22.301**	21.307**	26.423**	12.232**	12.178**
	D × V		3.872**	4.069**	5.088**	2.993*	3.539**
2018	SC704	6.3 (L)	167.07 ± 18.31a	386.50 ± 15.83d	249.48 ± 18.29a	7.92 ± 0.97b	4.95 ± 0.60d
		11.3 (H)	149.24 ± 21.02ab	330.95 ± 43.35e	150.03 ± 12.60d	7.56 ± 0.88bc	8.52 ± 0.99a
	ZD958	6.9 (L)	164.22 ± 8.55a	436.57 ± 16.71c	271.93 ± 5.60a	9.04 ± 0.51a	6.20 ± 0.35bc
		9.7 (M)	156.87 ± 15.32ab	286.25 ± 12.43f	208.28 ± 27.44b	8.35 ± 0.33ab	8.14 ± 0.32a
	XY335	7.0 (L)	144.00 ± 2.56ab	518.62 ± 41.65a	259.56 ± 27.94a	7.93 ± 0.30b	5.53 ± 0.21cd
		11.1 (H)	103.60 ± 13.50d	420.51 ± 15.66cd	175.27 ± 19.95cd	6.27 ± 0.42d	6.95 ± 0.47b
	DH618	7.5 (L)	134.95 ± 5.90bc	477.15 ± 25.51b	256.87 ± 13.60a	6.87 ± 0.08 cd	5.17 ± 0.06d
		9.9 (M)	118.70 ± 11.06cd	345.20 ± 18.39e	203.06 ± 10.52bc	6.87 ± 0.34 cd	6.77 ± 0.33b
	ANOVA						
	Density (D)		4.245 ns	141.952**	125.2**	3.491 ns	123.743**
	Variety (V)		11.282**	32.493**	6.676**	12.252**	6.802**
	D × V		3.894*	5.188*	2.322 ns	4.344*	10.994**

The mean values indicated within a column and the same site followed by different letters are significant differences at *p* < 0.05. L denotes plant density ≤ 7.5 plants m^−2^. M denotes plant density > 7.5 and ≤ 10.5 plants m^−2^. H denotes plant density > 10.5 plants m^−2^

### Sampling and measurements

#### Yield and yield components

Twenty ears from the center of four rows of each plot were collected and air-dried at physiological maturity to determine the number of kernels per plant and the 1000-kernel weight (TKW). To determine grain yield, plants at physiological maturity were manually harvested from a 12 m^2^ area in each plot, excluding the border rows. Maize grain and biomass yields were determined at 14% moisture content and dry weight, respectively.

#### Dry matter accumulation and leaf area

The aerial parts of three representative plants from each plot were taken in order to assess the accumulation of dry matter at the silking and maturity stages. The leaf area and the leaf area index were calculated as previously described (Xu et al. 2017). All samples were heat-treated at 105 °C for 30 min, then dried at 80 °C to constant weight to allow for an estimation of dry matter accumulation. The harvest index (HI) was calculated as the ratio of grain yield to the total biomass at the maturity stage (Curin et al. 2020).

#### Gas-exchange measurements

At the silking, filling, and maturity stages, the ear leaf of three representative maize plants were selected from each plot to measure the net photosynthetic rate (P_n_), the stomatal conductance (G_s_), the intercellular CO_2_ concentration (C_i_), and the transpiration rate (T_r_). These measurements were performed using a Li-Cor 6400 gas exchange system (Li-Cor Inc., Lincoln USA) equipped with an LED leaf chamber, with a steady photosynthetic photon flux density (PPFD) of 2000 μmol photons m^−2^ s^−1^ from 9:00 to 11:00 am on sunny, cloudless days. To determine dark respiration (*R*_d_) at night, gas exchange measurements were conducted from 11:00 pm until just before sunrise in the following day. A large leaf cuvette (2 cm × 6 cm) was used together with the LI-6400 gas analyzer to measure CO_2_ efflux. To determine dark respiration during the day, the leaves were dark-adapted for at least 30 min in advance (Niu et al. 2020).

#### Pigment measurements

The ear leaves were sampled at the silking, grain filling, and maturity stages. Leaf disks in the middle of the ear leaves (avoiding the midribs) were harvested using a hole puncher (area, 1.327 cm^2^) and frozen at − 80 °C for pigment determination. The amount of chlorophyll and carotenoids was measured spectrophotometrically, according to a previously published protocol (Lichtenthaler 1987). Briefly, milled leaf disks were incubated with 1 ml 100% acetone at 4 °C until the pigments were completely extracted. The mixture was then centrifuged at 10,000×*g* (4 °C) for 10 min, and the supernatants analyzed with a dual-beam spectrophotometer (Ultrospec 8000PC, Biochrom Ltd., Cambridge, England). All experimental procedures were conducted under low light conditions, and the samples were covered in order to minimize the degradation of chlorophyll as a result of light exposure. A total of three biological replicates were performed for each treatment.

#### Chlorophyll fluorescence at 77 K

For the 77 K measurements, leaf disks from the ear leaves (avoiding the midribs) were homogenized in solution (0.33 M sorbitol, 50 mM Hepes, pH 8, 1 mM MgCl_2_, and 2 mM Na_2_EDTA). The mixture was then filtered through two layers of Mira cloth and centrifuged at 10,000×*g* at 4 °C for 15 min. The sediment was resuspended in the same buffer, and the chlorophyll concentration was adjusted to 10 μg/ml. 77 K fluorescence emission spectra were recorded from 650 to 800 nm after excitation at 435 nm using the spectrofluorometer (F-7000, Hitachi, Japan) (Stockel and Oelmuller 2004). A total of three biological replicates were performed for each treatment.

#### Stomata analysis

Stomatal density and the stomatal aperture area were measured at the filling stage using nail polish impressions (Zheng et al. 2013). Specifically, colorless nail polish was evenly applied to the abaxial surface of the ear leaf in order to obtain a replica of the leaf surface. The impressions were then observed under a microscope (DM5500B, Leica Corp, Biberach, Germany) equipped with a digital camera (DFC300-FX, Leica Corp). The stomatal density was determined by counting the number of stomata in 18 fields of view per treatment. To determine the stomatal aperture area, nine stomata were randomly selected from different images captured under the microscope for each sample, four replications were performed for each treatment (totaling 36 stomata per treatment), and the stomatal aperture area was measured using ImageJ (NIH, Bethesda, MD, USA).

#### The ratio of red to far-red light

The ratio of red to far-red light was measured using a handheld spectrometer (HR-350, HiPoint Co., Ltd., Taiwan) at the ear and ground positions around noon at the silking and filling stages on a sunny, cloudless day. The device was kept vertically to the soil surface with the sensor backing against the plant and facing north, east, south, and west. The average of the four estimated values was used for analysis (Zhu et al. 2014). R/FR was calculated as the ratio of spectral irradiance measured in the bands at 655 to 665 nm (red) over the irradiance measured in the bands 725 to 735 nm (far red). A total of three independent replicates were performed for each treatment.

### Statistical analysis

Data were prepared and calculations were performed using Microsoft Excel 2016. An analysis of variance was performed with SPSS 21.0 (SPSS Institute Inc., US) in order to test for differences between treatments. The significance level was set at the 0.05 probability level. The statistical analysis for each year and growth stages was performed separately. The figures were generated using Origin Pro 8.0.

## Results

### Grain yield and yield components

The effects of density and variety on grain yield were all significant during the two growing seasons, whereas the density × variety interaction only had a significant effect on grain yield in the year 2017. The grain yield of five maize cultivars at medium planting density (105,000 plants ha^−1^) was greater than that observed at low density (75,000 plants ha^−1^) by 2.09–20.32% in both years (Table 1). Interestingly, increasing planting density from medium to high (105,000 to 135,000 plants ha^−1^) did not significantly change the grain yield of DH618, ZD985, and XY335, but caused a remarkable decrease in SC704 and LY66 (26.08% and 26.18%, respectively, compared to grain yield at medium density). Notably, the grain yield of SC704 and LY66 cultivated at high density was even lower than their yield under low density condition (Table 1). Comparing the five varieties, the highest grain yield was observed in LY66 at medium density, followed by DH618 in 2017.

Ear number increased with higher planting densities by 12.9–62.1% in all five maize cultivars in both years. In contrast, the kernel number per ear and the 1000-kernel weight (TKW) decreased with higher planting densities in both years. Importantly, both density and variety had significant impacts on kernel number per ear and the 1000-kernel weight, and the interaction between these factors had a dramatic effect on kernel number per ear while only marginally influencing the 1000-kernel weight in both years. LY66 and DH618 had relatively higher TKW than other cultivars, suggesting that TKW influences grain yield performance in response to changes in planting density. Additionally, the earless plant rate of SC704 cultivated at high density was up to 32.8% in 2017.

Increasing plant density significantly decreased the yield per plant, especially under high-density conditions (Table 2). Compared to other cultivars, LY66 and DH618 showed relatively higher yields per plant under the medium density cultivation condition. This is consistent with observations of the grain yield per plot and indicates different maize cultivars have different density tolerance. Overall, the increased yield per plant under medium and high-density conditions is important for further increasing the plant yield per area.

### Dry matter accumulation

Dry matter accumulation and distribution directly influence crop yield. Accordingly, we observed a significant influence of density, variety, and their interaction (density × variety), in the plant biomass measured at the silking and maturity stages. At the level of individual plants, the biomass at the silking and maturity stages declined as planting density increased in both years (Table 2). The dry matter per plant of DH618 cultivated at high density was lower compared to low density conditions by 21.68%. Similarly, we observed a proportional decrease of 34.09–40.81% for the ZD958, XY335, LY66, and SC704 varieties. This difference can help explaining the overall higher yield per plant obtained for DH618.

At the maize population level, total biomass accumulation increased as a function of planting density (Table 1). This tendency is consistent with the results obtained for yield, but is not exactly the same. For example, XY335 showed the highest total biomass at medium density conditions, but its overall yield was lower than both DH618 and LY66 (Table 1). Considering that only the biomass located to the seed contributes to yield, we further measured the harvest index (HI) across all varieties and density treatments. XY335 had the lowest HI of all maize cultivars. In contrast, DH618 and LY66 had the highest HI which, along with the observed higher total biomass accumulation in the latter two, suggests that biomass accumulation and its efficient distribution to seeds determines maize yield.

### Leaf area and leaf area index

According to previous studies, the leaf area and the leaf area index (LAI) are important parameters determining photosynthetic capacity (Sinclair et al. 2004), whereby we measured both of these parameters in order to understand the relationship between photosynthesis and maize yield. Our results demonstrated that LAI was significantly influenced by planting density and cultivar variety in both years. Specifically, the LAI at the silking stage increased significantly in all maize cultivars with increasing planting density (Table 2). In contrast, the leaf area per plant at the silking stage decreased as planting density increased (Table 2). Interestingly, no significant changes in leaf area were observed in ZD958 and XY335 (in 2017), and DH618 (in 2017 and 2018), between different planting densities. The leaf area per plant of the LY66 and SC704 cultivars grown at high density significantly decreased by 17.11% and 15.36% when compared to plants grown at low density in 2017. Moreover, the leaf area per plant and the LAI of DH618 were lower than in other cultivars, respectively, averaging 5.86 and 6.15 (Table 2). These results suggested that higher leaf area per plant and leaf area index do not necessarily improve maize yield.

### Gas exchange and dark respiration

**Fig. 2:**
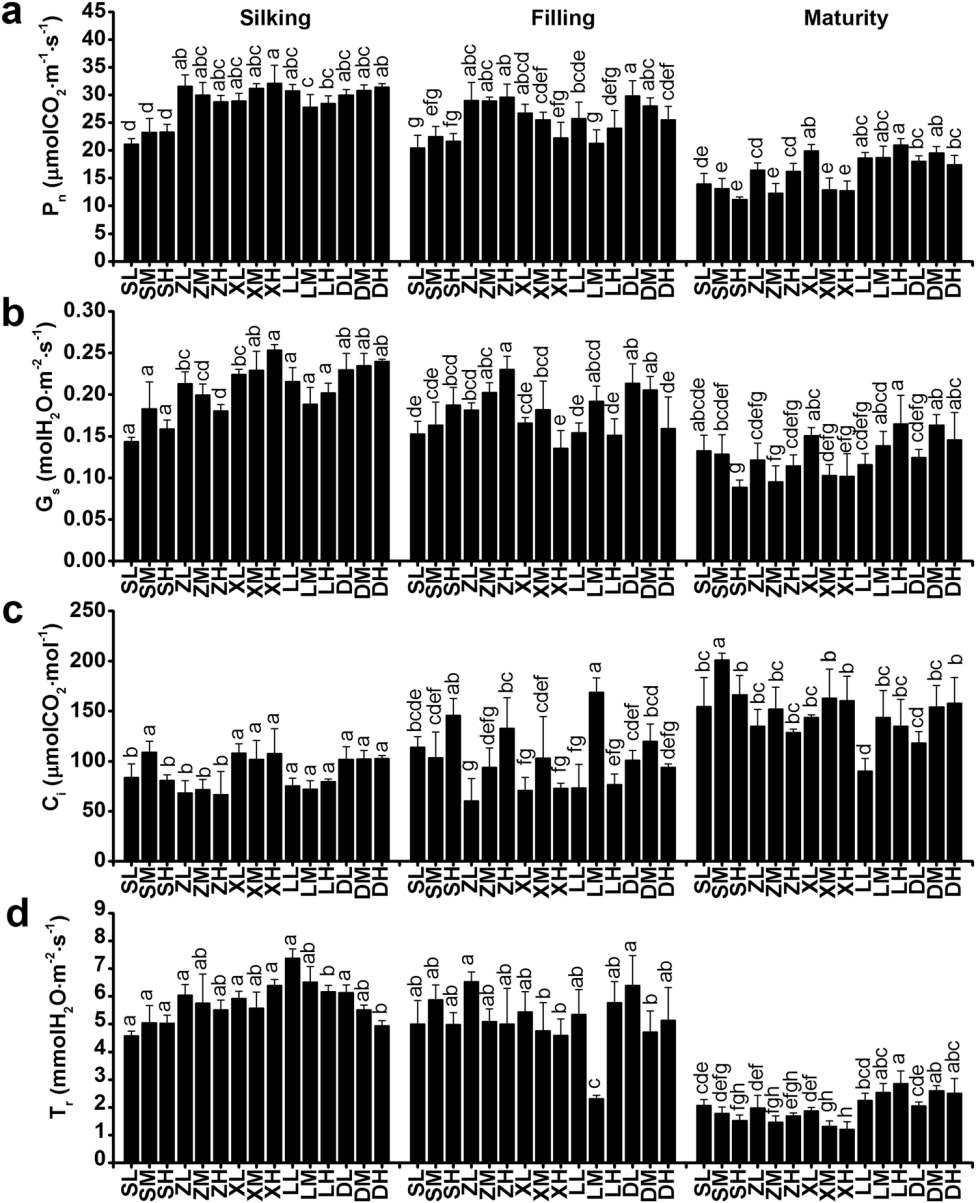
Photosynthetic characteristics of the ear leaf at the silking, filling, and maturity stages, as influenced by plant density in 2017. Different lowercase letters indicate significant differences (*p* < 0.05) between treatments. P_n_, net photosynthetic rate; G_s_, stomatal conductance; C_i_, intercellular CO_2_ concentration; T_r_, transpiration rate. SL, SC704 at low density; SM, SC704 at medium density; SH, SC704 at high density; ZL, ZD958 at low density; ZM, ZD958 at medium density; ZH, ZD958 at high density; XL, XY335 at low density; XM, XY335 at medium density; XH, XY335 at high density; LL, LY66 at low density; LM, LY66 at medium density; LH, LY66 at high density; DL, DH618 at low density; DM, DH618 at medium density; DH, DH618 at high density

**Fig. 3:**
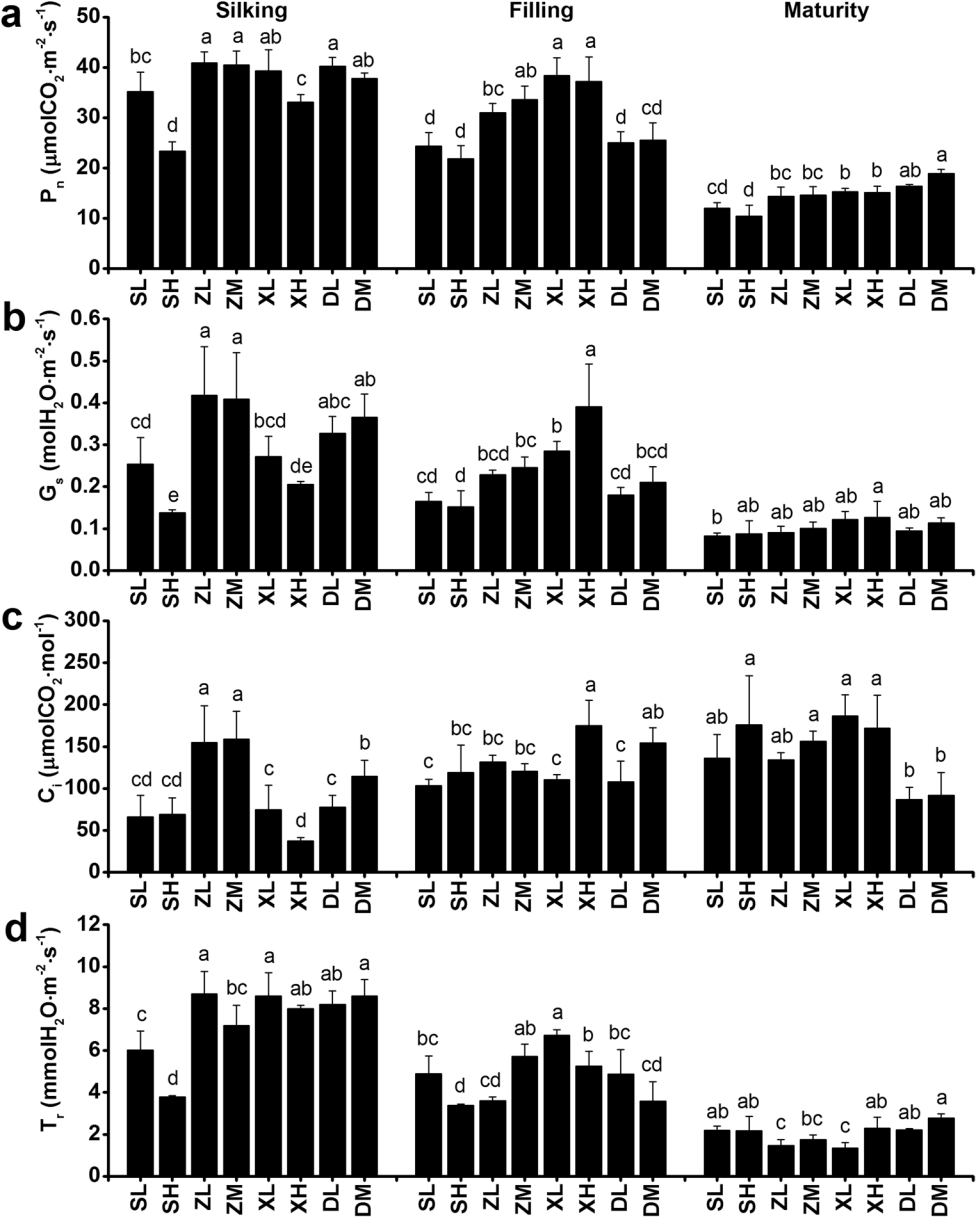
Photosynthetic characteristics of the ear leaf at the silking, filling, and maturity stages, as influenced by plant density in 2018. Different lowercase letters indicate significant differences (*p* < 0.05) between treatments. P_n_, net photosynthetic rate; G_s_, stomatal conductance; C_i_, intercellular CO_2_ concentration; T_r_, transpiration rate. SL, SC704 at low density; SM, SC704 at medium density; SH, SC704 at high density; ZL, ZD958 at low density; ZM, ZD958 at medium density; ZH, ZD958 at high density; XL, XY335 at low density; XM, XY335 at medium density; XH, XY335 at high density; LL, LY66 at low density; LM, LY66 at medium density; LH, LY66 at high density; DL, DH618 at low density; DM, DH618 at medium density; DH, DH618 at high density

Photosynthetic assimilation during post-silking is a critical determinant of grain yield. In order to better understand the changes in photosynthesis at different developmental stages, we measured the photosynthetic rate (P_n_) of ear leaves at the silking, filling and maturity stages in the field. Our measurements showed that P_n_ and *T*_r_ decreased significantly at the maturity stage in all maize cultivars in both 2017 and 2018. When compared to other cultivars, DH618 and LY66 had higher P_n_ and T_r_ at the maturity stage, which means that the former slowly declined in both cultivars during the filling stage, maintaining a relatively high photosynthetic activity at the maturity stage (Figs. 2, 3). During the early reproductive stage, no significant differences in P_n_ were observed between four of the maize cultivars at the silking stage, which averaged 29.00–30.73 μmolCO_2_ m^−2^ s^−1^. During the silking and filling stages, the SC704 cultivar showed a 22.20–26.60% lower P_n_ compared to the remaining varieties. In 2017, the planting density did not significantly impact the P_n_ of the ear leaves during all three growing stages (Fig. 2a). However, in 2018, the increase in planting density led to a significant decrease in the P_n_ of SC704 and XY335 at the silking stage, despite no significant differences existed at the filling and maturity stages (Fig. 3a).

**Fig. 4:**
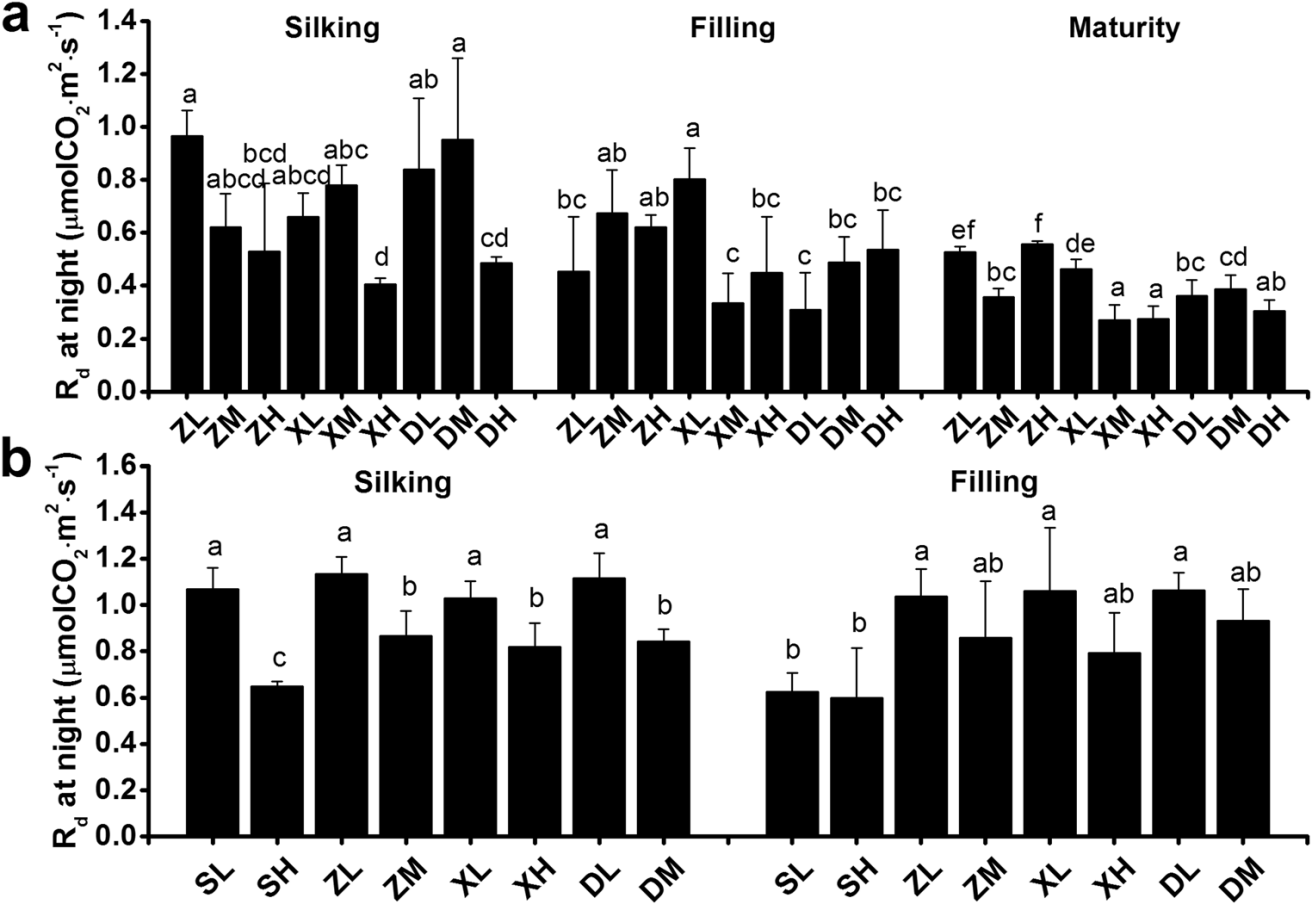
Dark respiration rate of maize ear leaves in 2017 (**a**) and 2018 (**b**). Different lowercase letters indicate significant differences (*p* < 0.05) between treatments. R_d_, dark respiration rate

Additionally, G_s_, C_i_, and T_r_ followed a similar trend to P_n_ for the different planting density treatments while causing a significant reduction in the *R*_d_ at night during the silking stage in both 2017 and 2018 (Fig. 4). At the filling and maturity stages, no significant differences were detected in the *R*_d_ across four cultivars (Fig. 4b). Finally, we also analyzed the stomatal status but found no changes on stomatal density and aperture area between treatments at the filling stage (Fig. S1).

### Pigment content

**Fig. 5:**
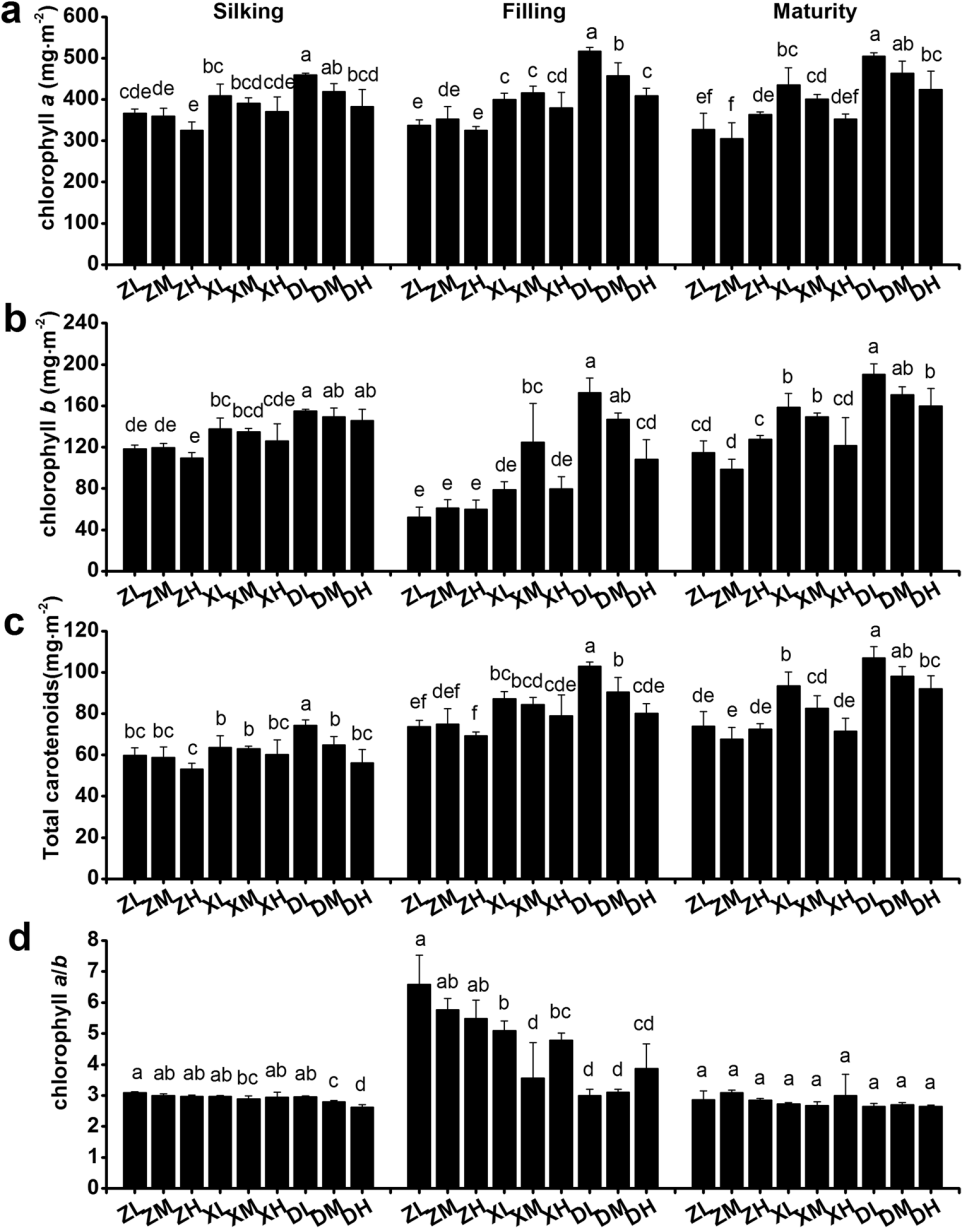
Pigment content and chlorophyll *a/b* ratio of the ear leaf during the growing season in 2017. **a** Amount of chlorophyll *a*; **b** Amount of chlorophyll *b*; **c** Amount of carotenoids; **d** chlorophyll *a/b* ratio. Different lowercase letters at the same growth stage indicate significant differences (*p* < 0.05) between treatments

Since we detected small differences in gas exchange and respiration rate between different planting densities, we opted to further analyze the amount and composition of pigments. Overall, the amount of chlorophyll *a*, chlorophyll *b*, and carotenoids decreased with increasing planting density during the growing season (Fig. 5a–c), and decreased in the following order for different cultivars: DH618 > XY335 > ZD958. The latter trend is similar to the observed grain yield in the year 2017 (Fig. 5, Fig. S2). The amount of chlorophyll *a* did not significantly change from the silking to the maturity stage, while the amount of chlorophyll *b* decreased after the silking stage and then increased at physiological maturity, resulting in a significant increase in the chlorophyll *a*/*b* ratio at the filling stage (Fig. 5d).

### 77 K chlorophyll fluorescence

**Fig. 6:**
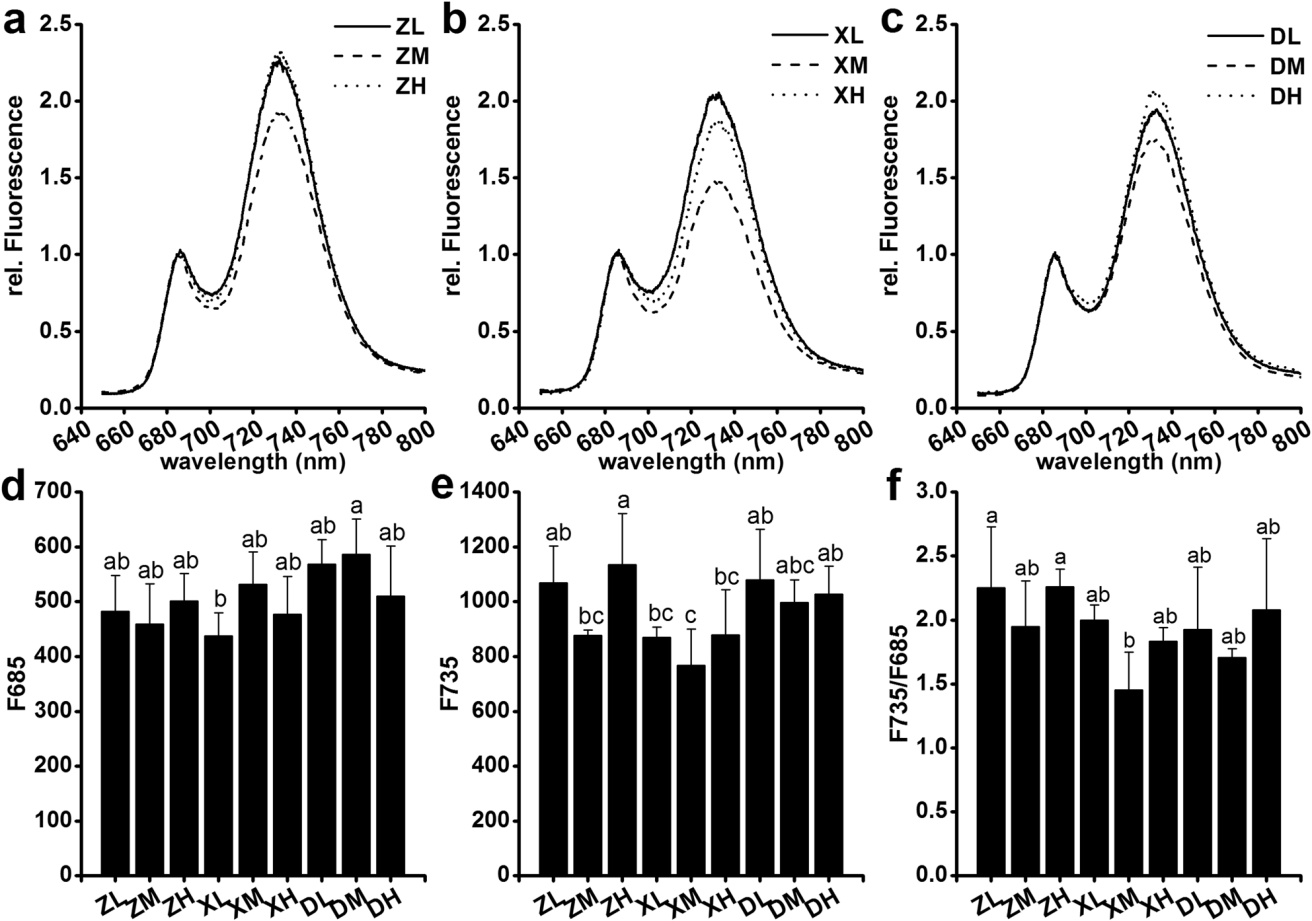
77K fluorescence emission spectra of the ear leaf at the filling stage in 2017. **a**–**c** 77 K fluorescence emission spectra after excitation at 435 nm. The chlorophyll concentration was adjusted to 10 μg/ml. The values were normalized to the emission at 685 nm. **d**–**f** 77 K fluorescence emission at 685 nm and 735 nm and the F735/F685 ratio. Different lowercase letters at the same growth stage indicate significant differences (*p *< 0.05) between treatments

To further examine the underlying reason behind differences in photosynthesis, we performed a fluorescence emission analysis at 77 K. All treatments showed similar 77 K fluorescence spectra, with emission peaks at 685 nm and 735 nm (Fig. 6a–c). The emission signal at 685 nm did not significantly differ between planting density treatments (Fig. 6d), nor did the F735/F685 ratio, an indicator of the excitation energy distribution between photosystems II and I (Fig. 6f). These data might explain the insensitivity of the photosynthetic rate in the ear leaves in response to increased planting density (Fig. 6, Fig. S3).

### The ratio of red to far-red light

Light quality changes as planting densities increase in a canopy. The light that passes through a canopy of leaves has a reduced red to far-red ratio (Holalu and Finlayson 2017). The ability of plants to adjust their morphology and physiology in response to light quality changes in natural environments might play an important role in plant development and grain yield. As shown in Fig. S4, the R/FR significantly decreased with increasing planting density on leaves located in the ear position at the silking stage in 2017 (except for DH618), and the filling stage in 2018. Additionally, the R/FR ratio on leaves located in ground positions decreased to a significant level in SC704 and XY335 under high-density conditions. However, the R/FR ratio of ZD958, LY66 and DH618 were not seemingly affected by different density treatments in leaves located in the ground position (Fig. S4a, c). A similar trend was observed in the ear position at the filling stage in 2017, and the silking stage in 2018. In general, the R/FR ratio of DH618 was not significantly affected by increasing planting density, indicating that a better shoot structure can minimize the competition for light and the effects caused by increased shading.

### Relationships of maize grain yield and photosynthetic capacity confirmed by genotypes released in different years

**Fig. 7:**
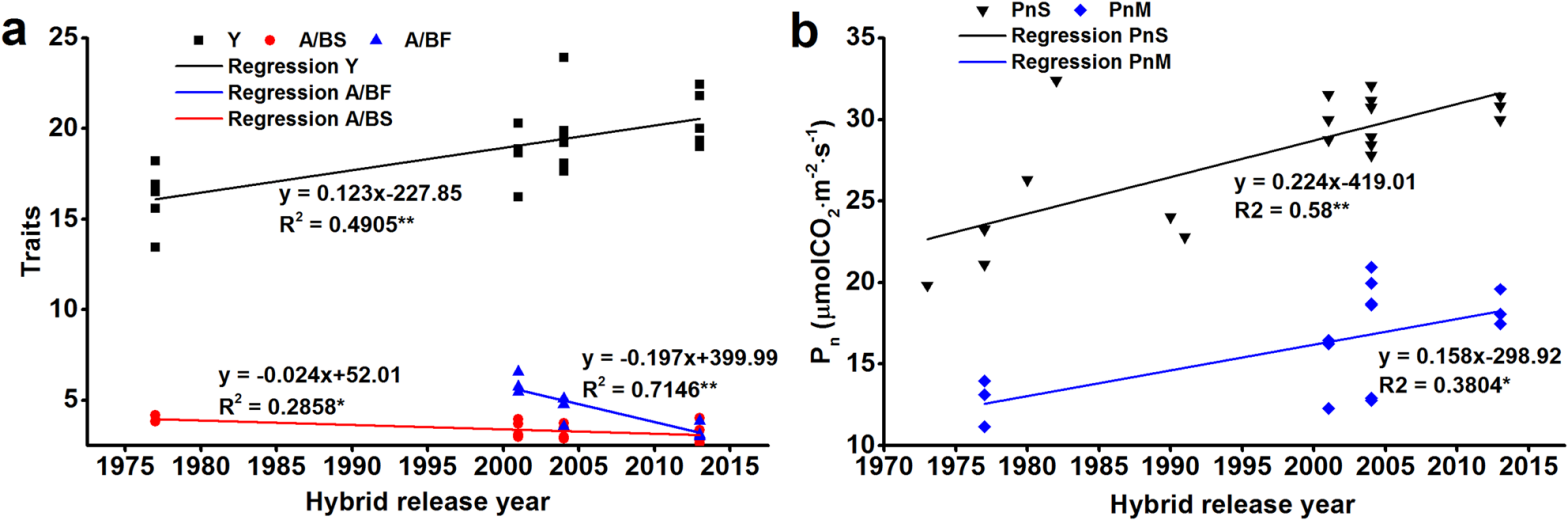
Trends in grain yield, net photosynthesis, and chlorophyll *a*/*b* ratio at the silking and filling stages over the hybrid release year. Y, grain yield; PnS, P_n_ at silking stage; PnM, P_n_ at maturity; A/BS, chlorophyll *a*/*b* at silking stage; A/BF, chlorophyll *a*/*b* ratio at filling stage. Data from previous studies, including maize varieties released between the 1970–1990 decades, were integrated into this figure (Zhang et al. 1992; Ding et al. 2006; Zhang et al. 2011; Li et al. 2015)

As shown in Fig. 7, a correlation analysis between different traits and the years of hybrid release showed that yield is positively correlated with the year of hybrid release (*R*^2^ = 0.49, *p* < 0.01), suggesting the possibility of cultivar improvement over the years. In contrast, the ratio of chlorophyll a/b at the silking (A/BS) and filling (A/BF) stages is negatively correlated with hybrid release year (*R*^2^ = 0.71, *p* < 0.01 and *R*^2^ = 0.29, *p* < 0.05). We further integrated data from other publications with our experimental data to confirm the relationship between P_n_ and hybrid release year. Our results clearly showed an increasing trend in the P_n_ from the 1970s to 2010s (*R*^2^ = 0.58, *p* < 0.01).

### Correlation analysis

**Fig. 8:**
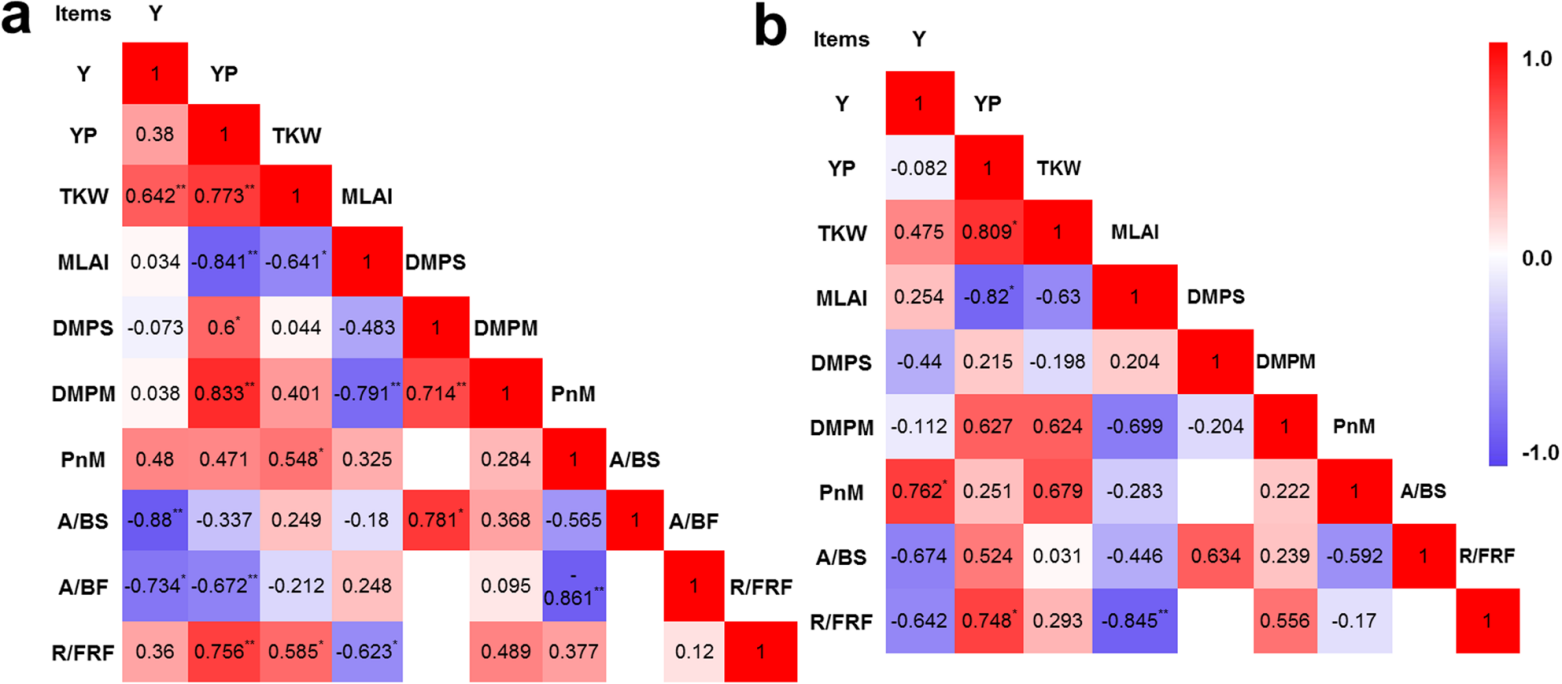
Relationship between grain yield and main physiological indices during the growing stage in 2017 (**a**) and 2018 (**b**). Y, grain yield; YP, yield per plant; TKW, 1000-kernel weight; MLAI, maximum LAI; DMPS, dry matter per plant at silking stage; DMPM, dry matter per plant at maturity; PnM, P_n_ at maturity; A/BS, chlorophyll *a*/*b* ratio at silking stage; A/BF, chlorophyll *a*/*b* ratio at filling stage; R/FRF, R/FR at filling stage. ∗, ∗∗ Significance at the 0.05 and 0.01 probability level, respectively

Figure 8 shows the results of a correlation analysis between all physiological parameters measured over the 2 years of field experiments. In both 2017 and 2018, the R/FR ratio at the filling stage has a significantly positive correlation with yield per plant (YP) and the 1000-kernel weight (TKW), which was stronger for the former. Additionally, the TKW has a positive correlation with grain yield (Y) and YP, while the dry matter per plant at the silking stage (DMPS) and the dry matter per plant at the maturity stage (DMPM) were both significantly correlated with YP in 2017, Moreover, DMPM showed a strong positive correlation with YP in 2018. Our analyses also showed that the maximum LAI (MLAI) was negatively correlated with YP but uncorrelated with Y. The P_n_ at maturity (P_n_M) was positively correlated with Y and TKW, but only significantly with the latter in 2017. Interestingly, significantly negative correlations were discovered between chlorophyll *a*/*b* and grain yield in both 2017 and 2018.

## Discussion

### Increase of maize yield potential by cultivar improvement

China’s population is expected to peak in 2033. Accordingly, grain production needs to increase by at least 35% over the next two decades to meet the demand of the rising population (Zhang 2011). Maize grain yield has continuously increased in the past decades due to advances in farming technology and maize hybrids. Increasing plant density is one important management practice for improving maize yield (van Ittersum and Cassman 2013; Zhang et al. 2020; Sher et al. 2017). However, there is a curvilinear response between grain yield per area and plant density in modern maize hybrids, demonstrating that yield will not increase indefinitely as plant density exceeds the optimal level (Mastrodomenico et al. 2018; Wei et al. 2019). In this study, we used five maize hybrid cultivars to analyze the physiological mechanisms that are potentially responsible for further increasing yield potential. We found the replacement of maize cultivars leading to enhanced tolerance to high planting density, higher yield per plant and higher grain yield per area (Table 1, Table 2 and Table S1).

The grain yield of the oldest cultivar, SC704, drastically decreased at high cultivation densities (135,000 plants ha^−1^), largely owing to a sharp decrease in HI that was associated with an increase in barren plants (earless plant rate up to 32.8%). The second oldest cultivar, ZD958, has a higher tolerance to high density its kernel number per ear and TKW are lower than more recent cultivars, which limits yield improvement. The cultivars XY335 and LY66 were both released in 2004, and the grain yield of the latter is the highest under medium planting density, but rapidly decreased at higher densities due to a decrease in the 1000-Kernel weight (16.74% decrease comparing to low density). Importantly, this indicates that density tolerance constitutes a bottleneck for the yield improvement of this cultivar. Unlike LY66, XY335 has good density tolerance and the highest biomass accumulation among all cultivars analyzed. However, its HI drastically decreased under both medium and high-density conditions. It was reported that modern commercial hybrids generally have enhanced tolerance to increased planting density compared to older maize hybrids (Echarte et al. 2000; Bernhard and Below 2020). DH618 is the newest cultivar of all analyzed and showed a stable high grain yield, the highest TKW and the least decrease in biomass per plant under the high-density condition (Tables 1, 2, Fig. 7 and Table S1). DH618 has higher grain yield resulting from higher 1000-kernel weight, which is related to the source-sink ratio established during the early grain filling stage (Brenda et al. 2006). Overall, our comprehensive comparison of yield components, biomass accumulation and distribution (HI) among five cultivars under three planting density conditions showed important indicators for improving yield potential of modern maize hybrids.

### High-yield cultivars have moderate leaf area with higher photosynthetic capacity and longer photosynthesis duration

To analyze the relationship between photosynthesis and grain yield, we systematically measured photosynthesis-related parameters in a time-course manner using five maize cultivars. According to previous research, the high-yield potential depends on appropriate canopy structure, including a favorable LAI that enables source supply (Boomsma et al. 2009; Wei et al. 2017). In this study, an expected increase in LAI was detected with increasing planting densities that facilitates an impressive capturing of solar radiation and leads to a concomitant increase in biomass (Table 1). However, intraspecific competition becomes stronger and light quality in the canopy changes with increased planting density (Yamazaki 2010). In present study, the R/FR ratio showed a close linear relationship with YP and a negative correlation with MLAI (Fig. 8). These observations support the finding that the lower LAI and decreased leaf area per plant in DH618 may be attributed to less mutual shading. Hence, the higher leaf area per plant and the leaf area index do not necessarily improve maize yield, and instead depend on other factors and circumstances. In this case, a moderate LAI could instead be more favorable for photosynthesis and yield formation.

The contribution of photosynthesis to grain yield improvement has been thoroughly debate for some years. Some reports suggested that an enhanced leaf photosynthetic capacity provides a basic avenue for improving maize yield (Long et al. 2015; Ren et al. 2016), while others argued for little correlation between increased photosynthesis and crop yields (Driever et al. 2014; Wu et al. 2018). Our results integrate previously reported data and showed that the photosynthetic rate increased synergistically with the replacement of older maize cultivars with modern varieties (from the 1970s to 2010s) (Figs. 2, 3 and 7), particularly when considering the P_n_ at late reproductivity stages. At the same time, we found a positive correlation between P_n_M and TKW and Y (Fig. 8), in agreement with previous work (Gai et al. 2017). The increased availability of carbohydrates is associated with an increase in the rate of dark respiration under elevated CO_2_ conditions (Li et al. 2013). We found that the *R*_d_ of SC704 during the night at the filling stage was lower than other cultivars (Fig. 4), which is consistent with a decrease in the P_n_. Moreover, increased dark respiration is associated with higher growth rates, as dark respiration provides ATP and converts carbohydrates to carbon (C) atoms that are needed for growth. Therefore, photosynthesis and respiration are highly coordinated to sustain plant growth. These observations showed that an improved photosynthetic efficiency might be the crucial contributor to higher grain yields.

It has been previously shown that high planting density reduced leaf stomatal density and net photosynthesis, leading to a decreased dry mass accumulation after silking (Yan et al. 2017; Wei et al. 2020). Here, we showed that the incremental increases in planting density do not result in significant differences in net photosynthesis (P_n_), stomatal conductance (G_s_), intercellular CO_2_ concentration (C_i_), and transpiration rate (T_r_) of the ear leaves. These results are not necessarily surprising, since changes on leaf traits depend on the plant species under consideration and the experimental treatment conditions. The literature shows that the P_n_, G_s_, T_r_, L_s_ and LAI first increase and then decreased with growing planting density in wheat (Fang et al. 2018). Here, we collected data at the beginning of the anthesis stage, which is the early reproductive stage, and also found that the P_n_, G_s_, and T_r_ decrease under high-density cultivation conditions (SC704 in 2018), but only at the silking stage. The negative effects of high-density cultivation on P_n_ disappeared at the filling and maturity stages in all tested maize cultivars in both 2017 and 2018. Hence, our time-course analysis includes more cultivars and provides a deeper, comprehensive view to changes in the P_n_ as a response to planting density. A study in cattails showed that the leaves from low planting density populations had higher stomatal density and index, a thicker mesophyll and a higher proportion of aerenchymal area, even though the evaluated plant developmental stage remains unknown (Corrêa et al. 2017). A further study showed that, compared to low density conditions, high-density cultivation decreased stomatal aperture area by 31.9% in maize, and that stomatal density was independent of the cultivar and the planting density at the filling stage (Niu et al. 2020). This is partly consistent with our results. In our case, however, neither stomatal density nor stomatal aperture area changed with density treatment at the filling stage. There are two possible reasons for these discrepancies. Firstly, we used a narrow-wide row planting pattern that provides more space and increases the amount of light reaching the ear leaf position. Secondly, the typical physiological responses (i.e., stomata, chlorophyll fluorescence and photosynthesis) to planting density are the result of plant competition for light resources (Wei et al. 2020; Postma et al. 2021). However, Xinjiang has adequate radiation during the growth period of maize, which is likely to reduce the amount of competition for light between different plants. The planting densities (75,000 plants ha^−1^, 105,000 plants ha^−1^ and 135,000 plants ha^−1^) did not significantly decrease the fluorescence of chlorophyll 77 K nor the amount of chlorophyll *a* and *b*, which is consistent with an unchanged stomatal status. Consequently, the planting densities used in our experiments did not have a significant effect on stomata controlling the supply of CO_2_ for photosynthesis (Fig. S1), the amount of pigments present nor the fluorescence associated with the photosynthetic processes (Figs. 5, 6, Fig. S3). Hence, it did not affect photosynthesis. Surprisingly, we found that DH618 and LY66 maintain a higher P_n_ at maturity at densities of 105,000 and 135,000 plants ha^−1^ (Figs. 2a and 3a). The amount of chlorophyll *b* in DH618 was higher than other cultivars, and it has been suggested that plants with higher chlorophyll *b* have slower leaf senescence (Janečková et al. 2019). It is thus possible that DH618 and LY66 may have slower leaf senescence during the reproductive period, consequently resulting in a higher 1000-kernel weight and grain yield (Kusaba et al. 2013).

### Physiological indicators for high-yield maize cultivars

Chloroplasts are the main photosynthetic organelle, and the amount of chlorophyll and composition support the biochemical energy production for the Calvin-Benson cycle by harvesting photons and transporting electrons, which is crucial to determine photosynthesis that accounts for more than 98% of the variation in maize primary production (Alton 2017; Luo et al. 2019; Gitelson et al. 2008). Previous studies provided compelling evidence that the total amount of chlorophyll is significantly correlated with yield-determining traits, including grain yield (Ghimire et al. 2015). In our study, DH618 had a higher chlorophyll amount than ZD958 and XY335, as well as a lower chlorophyll *a/b* ratio than the two other cultivars, particularly at the filling stage. As chlorophyll *a* helps converting energy of absorbed photons to chemical energy, and chlorophyll *b* is in charge of harvesting light (Björn et al. 2009; Kume et al. 2018), our results suggest that the light-harvesting antennae proteins are much more abundant than the photosystem core proteins in DH618, when compared to other cultivars. The higher amounts of light harvested in DH618 leaves produce higher photosynthetic efficiency and greater amounts of carbohydrates, leading to a higher yield. Notably, the chlorophyll *a/b* ratio at the silking (*R*^2^ = 0.77**) and filling stages (*R*^2^ = 0.54*) had significantly negative correlations with yield in 2017 (Fig. 8a), and a strong negative association was also observed in 2018 (Fig. 8b). Moreover, the chlorophyll *a*/*b* ratio showed a negative correlation with hybrid release year (Fig. 7), whereby we assume that a lower chlorophyll *a/b* ratio could be used as a new indicator for selecting high-yielding cultivars.

## Conclusions

We showed that the difference in maize yield potential in our study is associated with differences in the photosynthetic capacity of different maize cultivars. The cultivars with higher yields and tolerance to planting density exhibited a higher photosynthetic capacity, a longer duration of photosynthesis, a higher amount of chlorophyll with a lower chlorophyll *a/b* ratio, a moderate leaf area, and LAI. Additionally, maize photosynthesis, dark respiration, stomatal density, and aperture showed no significant changes with increasing planting density. Therefore, we propose that photosynthesis capacity is a primary determinant of maize yield and that both of these factors are not necessarily associated with other physical and physiological traits.

## Supplementary Information

Below is the link to the electronic supplementary material.

Supplementary material 1 (TIFF 61 kb) **Fig. S1** Stomatal density and aperture area of the ear leaf under different densities at the filling stage of 2018. Different lowercase letters in the same growth stage denote significant differences (*p* < 0.05) between treatments

Supplementary material 2 (TIFF 55 kb) **Fig. S2** Total chlorophyll amount of the ear leaf under different densities during the growing season of 2017. Different lowercase letters in the same growth stage denote significant differences (*p* < 0.05) between treatments

Supplementary material 3 (TIFF 58 kb) **Fig. S3** F_v_/F_m_ of the ear leaf under different densities during the growing season of 2017. Different lowercase letters in the same growth stage denote significant differences (*p* < 0.05) between treatments

Supplementary material 4 (TIFF 172 kb) **Fig. S4** The ratio of red to far-red light (R/FR) on ear and ground position, respectively, at silking (a, c) and filling (b, d) under different densities in 2017 (a-b) and 2018 (c-d). Different lowercase letters in the same growth stage denote significant differences (*p* < 0.05) between treatments

Supplementary material 5 (DOCX 16 kb) **Table S1** The time of release, highest yield, highest-yielding year and planting density of the five maize hybrids used in this study
