# Supplementary material for: Improving photosynthesis to increase grain yield potential: an analysis of maize hybrids released in different years in China
Source: Photosynth Res. 2021 May 25;150(1-3):295–311. doi: 10.1007/s11120-021-00847-x (PMC8556214; doi:10.1007/s11120-021-00847-x)
Supplement: Supplementary file 5 — Supplementary material 5 (DOCX 16 kb) Table S1 The time of release, highest yield, highest-yielding year and planting density of the five maize hybrids used in this study [file 11120_2021_847_MOESM5_ESM.docx]

**Table S1** The time of release highest yield, highest-yielding year and planting density of the five maize hybrids used in this study

| Cultivar | Time of release | Year | Highest yield (Mg·ha^-1^) | planting density (10^4^·ha^-1^) |
| --- | --- | --- | --- | --- |
| SC704 | 1977 | 2007 | 16.3 | 9.0 |
| ZD958 | 2000 | 2009 | 20.4 | 12.0 |
| XY335 | 2004 | 2013 | 20.8 | 10.5 |
| LY66 | 2004 | 2012 | 21.2 | 13.5 |
| DH618 | 2013 | 2013 | 22.7 | 13.5 |
